# Prevalence of dental caries in pregnant Colombian women and its associated factors

**DOI:** 10.1186/s12903-023-03419-8

**Published:** 2023-10-24

**Authors:** Juliana Velosa-Porras, Nelcy Rodríguez Malagón

**Affiliations:** 1 Ph.D. Program in Clinical Epidemiology, Department of Clinical Epidemiology and Biostatistics, Pontificia Universidad Javeriana, Bogotá, Colombia; 2 MPH, Biostatistics, Department of Clinical Epidemiology and Biostatistics, Pontificia Universidad Javeriana, Bogotá, Colombia

**Keywords:** Oral health, Pregnant women, Dental caries, DMF index, Social determinants of health

## Abstract

**Objective:**

To identify the prevalence of dental caries in pregnant women in the Colombian population and its association with the medical history and social determinants, based on data from the fourth National Oral Health Survey (ENSAB IV).

**Materials and methods:**

A total of 1,047 pregnant women from different areas of Colombia were evaluated. A dental evaluation was performed using a flat oral mirror and blunt-tipped probe (World Health Organization, 2007). For diagnosis of the dental condition, the DMFT index was used. A negative binomial regression analysis was performed to evaluate the association between social determinants and the DMFT index.

**Results:**

The results of this national study show a 59% prevalence of caries in this population. Regarding the experience of caries, 89.9% of pregnant women showed having had caries.

**Conclusions:**

The results of this national study on pregnant women show a high prevalence of dental caries. The women’s level of education is an important factor associated with dental caries and filled teeth, so the role of oral health education and dental check-ups are important.

**Clinical relevance:**

The findings of this study show the oral health situation of pregnant women, with a high prevalence of dental caries. This leads to the development and strengthening of oral health education strategies that empower pregnant women in their care. In addition, dental checkups during pregnancy should be implemented and reinforced to prevent and treat oral pathologies and thus prevent complications during this stage.

**Supplementary Information:**

The online version contains supplementary material available at 10.1186/s12903-023-03419-8.

## Introduction

Pregnancy is a physiological state in which local and general changes occur, due to endocrine alterations and the mechanical effect of fetal development, which produce important changes in the body of the pregnant woman that become more significant as the pregnancy progresses [1]. During this period, oral and periodontal tissues are altered due to changes in saliva production that alter salivary pH (decreasing it and affecting the neutralizing capacity), and to modifications in the microbiome of the oral cavity, which constitute factors that affect the tissues of the oral cavity [2]. These circumstances make pregnant women susceptible to the development of oral conditions such as dental caries.

According to the World Health Organization (WHO), dental caries is one of the most prevalent preventable oral diseases worldwide and can be treated in its initial stages [3]. In most countries with low and middle incomes per capita, the prevalence of this disease continues to increase mainly due to poor access to health services and living conditions. Dental caries is the result of the formation of biofilm on the surface of teeth, which converts the free sugars contained in the food into acids that demineralize the mineralized structures of the tooth [4].

In Colombia, a middle-income country, according to the III National Oral Health Study conducted in 1998 [5], the prevalence of caries evaluated through the DMF (Decay, Missing, and Filled) index reached 76% in the adult population. In 2014, the IV National Oral Health Study was carried out. [6] It was observed that this disease continues to be one of the main oral diseases in the country, although the prevalence of the DMF index decreases with age until it reaches 43.4%.

Oral diseases, in general, are painful and, in some situations, generate disability in the entire population, including pregnant women. Socioeconomic factors, systemic status, lack of resources, barriers to access, and lack of priority by the Government concerning oral health problems may put pregnant women at greater vulnerability concerning this condition. This study aimed to identify the prevalence of dental caries in pregnant women in the Colombian population based on data from the fourth National Oral Health Study (ENSAB IV). Additionally, the potential association between dental caries in pregnant women and reported medical history and social determinants was explored.

## Methodology

### Study population

The National Oral Health Study [7] is a periodic survey designed to assess the health, disease and oral care conditions of the Colombian population. The last survey conducted called the fourth study collected information between 2013 and 2014 and used a representative sample of the Colombian population that evaluated 20,493 people, of whom 1,050 were pregnant. This study used the database of this survey. The sample design of the survey was stratified, multistage and with elements detailed in another publication [6]. Pregnant women were included in all the blocks or cartographic segments of the dispersed rural area included in the sample, thus generating a representative sample of pregnant women in Colombia.

The study was approved by ethics committee of the Pontificia Universidad Javeriana (Comité de Investigación y Ética agreement # 55, 2014). All methods were carried out in accordance with the guidelines and regulations established in the Declaration of Helsinki. Written Informed consent was obtained from all participants/legal guardian.

### Measurement and quality control

The survey consisted of 75 questions: the first 15 questions related to socio-economic aspects, the next 37 questions corresponded to lifestyles and oral health, and the last 23 questions inquired about behaviors and habits in oral health. In addition, a questionnaire was designed for pregnant women with 21 questions.

In addition to the survey, a clinical examination was performed by 24 teams composed of a coordinator, examining dentist, interviewer, and assistant.

All pregnant women underwent a clinical oral examination with the support of a portable dental unit and a photophore. The examiner had a flat-mouth mirror and a blunt-tipped probe (World Health Organization, 1997). For the diagnosis of the dental condition, the Decay, Missed, Filled teeth (DMFT) index was used. The registration was performed tooth by tooth, except for the third molar. The prevalence of caries was evaluated as the proportion of people who at the time of the exam had dental caries lesions over the total number of pregnant women in the sample. The caries experience is the proportion of people who at the time of the exam had evidence of having suffered caries (decayed, filled, or missing teeth) over the total number of pregnant women. The examining dentists received a theoretical course of 52 h and performed practical exercises using dental models. For the evaluation of the degree of interexaminer and intraexaminer agreement, 45 subjects were evaluated, and a Kappa index was obtained (interexaminer: 0.7; intraexaminer: 0.9) [6].

The quality of the survey data was mesh validated and incorporated into the capture applications throughout the field operation, which sent alerts at the time of entering improbable or inconsistent data to guarantee the consistency of the information, relationship of intra-form questions, and clinical exams. Additionally, the information was verified through random telephone calls. The corresponding validations were applied to each database, and inconsistencies in data entry were identified with markers. The data from the clinical exams were reviewed by validators to verify them after the fieldwork was completed.

For the analysis of the variables that represent the demographic characteristics, the context within which the region was located (territorial organization) and the health regime was considered (In Colombia in 1993, a reform of the health system was conducted to improve access to health services through mandatory universal insurance. The system has two types of insurance: contributory insurance, financed by payroll contributions, and subsidized insurance for the poorest population, which is financed by taxes (see Supplementary Table 1).

Descriptive bivariate analysis was performed to establish the association of the DMF index (tooth) and for each of its components using the median, Kruskal–Wallis, and chi-square tests. To determine the factors associated with the DMFT index, Decay, Missing, and Filled, a negative binomial regression model was used, because of the plenty number of zeros present in the data. The interaction was evaluated using the backward method with the chunk test strategy. No evidence of the presence of interactions was found. The presence of the confounding effect was determined by the change in the CR when compared with the complete model. Accuracy was assessed by identifying the CIs of the CR with lower amplitude. The AIC and BIC were used to establish the best model. The best model was established when the potential confounders were controlled, and the most parsimonious model was reached. The final association was explored with the calculation of the CR and the objective CIs of the modeling. The analysis was performed in Stata v14.0.

## Results

The population evaluated was 1,050 pregnant women, of which two patients had medical conditions that precluded the clinical examination. Of the remaining 1,048 women, 1 woman had no teeth, leaving a definitive sample of 1,047. The largest age group was 15 to 24 (51.2%), and 38.1% of subjects were in the second trimester of pregnancy.

The characteristics of the pregnant women are described in Table 1. This group was composed of women with very low monthly household incomes, married or living with a partner, unemployed, and belonging to the subsidized insurance population. Although a high number of pregnant women were undergoing medical treatment for prenatal care, a small number of them were not referred for dental consultation, and of those who were referred, very few attended. A high number of them reported using a toothbrush and brushing their teeth three times a day, but only 4 mentioned using toothpaste with the toothbrush.

**Table 1:** Sociodemographic characteristics and dental lifestyle among pregnant women (N = 1047)

	Variables		N (%)
**Demographic variables**	Decay	At least one tooth	618 (59)
	Filled	At least one tooth	743 (70.9)
	Missing	At least one tooth	406 (38.7)
	Age group	15–24	537 (51.2)
		25–34	413 (39.4)
		35–45	97 (9.2)
	Trimester of pregnancy	First	293 (27.9)
		Second	399 (38.1)
		Third	355 (33.9)
**Context**	Region	Atlantica	208 (19.8)
		Oriental	152 (14.5)
		Central	148 (14.1)
		Pacifica	175 (16.7)
		Bogotá	186 (17.7)
		Orinoquia - Amazonia	178 (17)
	Marital status	Married or living with a partner	774 (73.9)
		Not married or not living with a partner	273 (26)
	Race / Ethnicity	White	277 (26.4)
		Mongrel	424 (40.5)
		Black / Brown	125 (11.9)
		Other ethnicities	78 (7.4)
		don’t know / not defined	143 (13.6)
	Health Insurance System	Contributory	399 (38.1)
		Subsidiary	648 (61.8)
	Household Income	< 1 monthly salary	442 (42.2)
		1–2 monthly salaries	379 (36.2)
		> 2 monthly salaries	226 (21.5)
	Education Level	Primary	137 (13)
		Secondary school	569 (54.3)
		Technical	228 (21.7)
		University degree or more	113 (10.7)
	Job	Yes	335 (32)
		No	712 (68)
	Water supply	Aqueduct with a constant supply	732 (69.9)
		Aqueduct with intermittent supply	188 (17.9)
		Other water sources	127 (12.1)
	Living area	Rural	150 (14.3)
		Urban	897 (85.6)
	Type of housing	House	617 (58.9)
		Apartment	347 (33.1)
		Other types of housing	83 (7.9)
	Number of household members	Mean (S.D.)	4.35 (2.05)
	Last dental visit	Never	27 (2.6)
		More than 2 years	88 (8.4)
		Between 6 months and < 1 year	108 (10.3)
		Less than 6 months ago	817 (78.5)
**Individual lifestyles and behaviors**	Reasons for last dental visit	Never	27 (2.5)
		Emergency	78 (7.4)
		Treatment	242 (23.1)
		Prevention / Control	278 (26.5)
		Antenatal care	422 (40.3)
	Dental care place	Never	27 (2.5)
		Health center	86 (8.2)
		EPS	807 (77)
		Private practice	127 (12.1)
	Referral to the dentist	No	367 (35)
		Referred / no appointment	78 (7.4)
		Referred / appointment	602 (57.5)
	Trimester of referral	No referral/no appointment	445 (42.5)
		First	351 (33.5)
		Second	216 (20.6)
		Third	35 (3.3)
	Currently undergoing medical treatment	Yes	753 (71.9)
		No	294 (28)
	Type of medical treatment	No	294 (28)
		Antenatal care	704 (67.2)
		Other	49 (4.6)
**Dental lifestyle**	Toothbrushing frequency	No	1 (0.1)
		Once a day	68 (6.4)
		2 / day	304 (29)
		≥3 day	674 (64.3)
	Amount of toothpaste	No	1 (0.1)
		¼ brush	120 (11.4)
		½ brush	364 (34.7)
		¾ brush	329 (31.4)
		All brush	233 (22.2)
	Dental floss frequency	No	590 (56.3)
		Not every day	168 (16)
		Once a day	178 (17)
		Twice or more a day	111 (10.6)
	Oral hygiene items	Toothbrush	1020 (97.4)
		Toothbrush and toothpaste	3 (0.29)
		Toothbrush, toothpaste, dental floss and mouthwash	1 (0.1)
		No toothbrush	23 (2.2)

Table 2 presents the prevalence and caries experience in the population, stratified by each of the variables studied. The prevalence of caries for this population was 59%, which means that at the time of the examination, more than half of the pregnant women had a caries lesion, being very similar in the age groups 15–24 years and 25–34 years. Regarding the caries experience, 89.9% of pregnant women have had caries at some point in their lives.

**Table 2:** DMFT according to sociodemographic, context, individual lifestyles and behaviors, and dental care lifestyles of pregnant women

Variables				DMF		D		F		M	
		Prev (%)	Caries Exp (%)	median	IQR	median	IQR	median	IQR	median	IQR
Age group	15–24	29.3	43.4	4	1–7	1	0–2	1	0–4	0	0–0
	25–34	24.2	37.5	7	4–11	1	0–3	4	1–7	1	0–2
	35–45	5.5	9	11	7–15	1	0–3	5	2–9	2	0–4
	*p*			0.001*		0.233		0.000*		0.000*	
Trimester of pregnancy	First	17	25	5	2–10	1	0–2	3	0–7	0	0–1
	Second	23	34	6	3–9	1	0–2	3	0–6	0	0–2
	Third	20	31	6	2–9	1	0–3	2	0–6	0	0–1
	*p*			0.732		0.716		0.207		0.161	
Region	Atlantica	15	17.9	5	2–8	2	0.5-4	1	0–3	0	0–2
	Oriental	9.1	13.4	6	4-10.5	1	0–2	4	1–7	0	0–2
	Central	6.1	12.3	4	2–9	0	0–1	3	1–6	0	0–1
	Pacifica	8.1	13.4	5	1–9	0	0–2	2	0–6	0	0–1
	Bogotá	10.7	16.8	6	3–9	1	0–2	3	1–6	0	0–1
	Orinoquia - Amazonia	10	15.8	7	3–11	1	0–2	3	1–7	0	0–2
	*p*			0.004*		0.000*		0.000*		0.004*	
Marital Status	Married or living with a partner	43.4	66.5	6	3–9	1	0–2	3	0–6	0	0–2
	Not married or not living with a partner	15.5	23.3	5	2–9	1	0–2	2	0–5	0	0–1
	*p*			0.124		0.645		0.159		0.287	
Race / Ethnicity	White	15.3	24	6	3–10	1	0–2	3	0–6	0	0–1
	Mongrel	23	36.9	6	3–9	1	0–2	3	1-6.5	0	0–1
	Black / Brown	7.3	9.6	4	1–6	1	0–3	0	0–3	0	0–1
	Other ethnicities	5.2	6.9	8.5	4–12	1	0–4	3	1–7	1	0–3
	don’t know / not defined	8	12.2	5	2–9	1	0–2	3	0–5	0	0–1
	*p*			0.000*		0.059		0.000*		0.001*	
Currently undergoing medical treatment	Yes	40.4	63.8	6	2–9	1	0–2	3	0–6	0	0–2
	No	18.6	26	5	3–10	1	0–2	3	0–6	0	0–1
	*p*			0.603		0.023*		0.989		0.029*	
Type of medical treatment	No	18.7	26.1	5	3–10	1	0–2	3	0–6	0	0–1
	Antenatal care	37.8	59.4	6	2–9	1	0–2	3	0–6	0	0–2
	Other	2.5	4.3	5	4–8	1	0–2	3	1–5	0	0–1
	*p*			0.684		0.049*		0.833		0.091	
Health Insurance System	Contributory	18.4	34.9	6	3–9	0	0–2	4	1–7	0	0–1
	Subsidiary	40.6	54.9	5	2–9	1	0–3	2	0–5	0	0–2
	*p*			0.272		0.000*		0.000*		0.495	
Household Income	< 1 monthly salary	26.5	37.8	5	2–9	1	0–3	2	0–5	0	0–2
	1–2 monthly salaries	20.9	33	6	3–9	1	0–2	3	0–6	0	0–1
	> 2 monthly salaries	11.5	19	6	2–9	1	0–2	3	0–7	0	0–1
	*p*			0.838		0.001*		0.001*		0.019*	
Education Level	Primary	9.1	11.9	6	3–12	2	0–4	1	0–4	0	0–3
	Secondary school	33.5	48.2	5	2–9	1	0–2	2	0–5	0	0–1
	Technical	12.3	19.8	5	3–9	1	0–2	3	1–6	0	0–1
	University degree or more	4	9.8	6	3–9	0	0–1	5	1–7	0	0–1
	*p*			0.392		0.000*		0.000*		0.031*	
Job	Yes	17.9	29.7	6	3–10	1	0–2	4	1–7	0	0–2
	No	41	60.1	5	2–9	1	0–2	2	0–5	0	0–1
	*p*			0.003*		0.332		0.000*		0.003*	
Water supply	Aqueduct with a constant supply	39.3	63.4	6	3–9	1	0–2	3	0–6	0	0–1
	Aqueduct with intermittent supply	12.7	16.2	5.5	2.5-9	2	0–3	2	0–4	0	0–1
	Other water sources	6.9	10.2	5	2–9	1	0–3	1	0–5	0	0–2
	*p*			0.248		0.000*		0.002*		0.865	
Living area	Rural	9.4	12.7	6	2–9	1	0–3	1.5	0–5	0	0–2
	Urban	49.5	77.1	6	3–9	1	0–2	3	0–6	0	0–1
	*p*			0.914		0.127		0.401		0.433	
Type of housing	House	34.1	53.5	6	3–10	1	0–2	3	0–6	0	0–2
	Apartment	19	29.7	6	3–9	1	0–2	3	0–6	0	0–1
	Other type of housing	5.9	6.7	4	2–9	1	0–3	0	0–3	0	0–2
	*p*			0.154		0.086		0.001*		0.884	
Last dental visit	Never	1.8	1.8	2	0–4	2	0–4	0	0–0	0	0–0
	More than 2 years	5.9	7.2	4	2–7	1	0–3	1	0–3	0	0–1
	Between 6 months and < 1 year	6.3	9.1	6	2–9	1	0–2	2	0-6.5	0	0–2
	Less than 6 months ago	44.6	71.1	6	7	1	0–2	3	1–6	0	0–2
	*p*			0.000*		0.066		0.000*		0.000*	
Reasons for last dental visit	Never	1.8	1.8	2	0–4	2	0–4	0	0–0	0	0–0
	Emergency	5.3	6.8	6	3–8	1	0–3	2	0–4	1	0–2
	Treatment	13.7	22	7	3–11	1	0–2	4	1–7	0	0–2
	Prevention / Control	15.7	23.1	5	2–9	1	0–2	2	0–5	0	0–1
	Antenatal care	22.3	36	5	3–9	1	0–2	3	0–6	0	0–2
	*p*			0.001*		0.128		0.000*		0.000*	
Dental care place	Never	1.8	1.8	2	0–4	2	0–4	0	0–0	0	00-
	Health Center	4.9	7.4	5	2–7	1	0–2	2	0–4	0	0–1
	EPS	46	69.3	6	3–9	1	0–2	3	0–6	0	0–2
	Private practice	6.2	11.2	6	3–10	1	0–2	3	1–7	0	0–1
	*p*			0.003*		0.218		0.000*		0.001*	
Referral to the dentist	No	22.5	31	5	2–10	1	0–3	2	0–6	0	0–1
	Referred / no appointment	4.3	6.7	6	3–9	1	0–3	3	0–6	0	0–1
	Referred / appointment	32	52	6	3–9	1	0–2	3	0–6	0	0–2
	*p*			0.869		0.064		0.337		0.378	
Trimester of referral	No referral/no appointment	26.9	37.8	5	2–9	1	0–3	2	0–6	0	0–1
	First	19.4	30.8	6	3–10	1	0–2	3	1–6	0	0–2
	Second	10.5	18.1	5	2–8	1	0–2	3	0–5	0	0–1
	Third	2.1	3.1	6	2–7	1	0–2	3	0–6	0	0–2
	*p*			0.016*		0.042*		0.148		0.279	
Toothbrushing frequency	No	0.1	0.1	3	3–3	1	1–1	0	0–0	2	2–2
	Once a day	5	6	6	3–10	2	1–3	2	0–5	0	0–2
	2 / day	18.7	25.8	5	2–9	1	0–3	2	0–5	0	0–2
	≥ 3 day	35.2	57.8	6	2–9	1	0–2	3	0–6	0	0–1
	*valor p*			0.215		0.027*		0.312		0.190	
Amount of toothpaste	Less than ¼ brush	6.49	10.5	7	2–10	1	0–2	3	1–7	0	0–2
	½ brush	20.4	31.9	6	3–10	1	0–2	3	1–6	0	0–1
	¾ brush	18.3	27.4	6	2–9	1	0–2	2	0–6	0	0–1
	All brush	13.7	20.0	5	2–8	1	0–2	2	0–5	0	0–1
	*p*			0.058		0.884		0.015*		0.852	
Dental floss frequency	No	37.8	49.7	5	2–9	1	0–3	1	0–4	0	0–2
	Not every day	8.2	14.6	6	3–10	1	0–2	4	1–7	0	0–1
	Once a day	8.4	15.8	6	4–9	0	0–2	4	1–7	0	0–1
	Twice or more a day	4.6	9.7	6	3–10	0	0–1	4	2–7	0	0–1
	*p*			0.008*		0.000*		0.000*		0.561	
Oral hygiene items	Toothbrush	57.4	87.5	6	3–9	1	0–2	3	0–6	0	0–1
	Toothbrush and toothpaste	0.19	0.3	9	4–9	1	0–9	3	0–7	0	0–2
	Toothbrush, toothpaste, dental floss and mouthwash	0.1	0.1	8	8–8	1	1–1	7	7–7	0	0–0
	No toothbrush	1.3	2	4	1–8	1	0–2	2	0–6	0	0–1
	*p*			0.199		0.730		0.671		0.516	
Number of household members	Mean (S.D.)			4.35 (2.05)		1.72 (2.37)		3.61 (3.84)		1.05 (2.10)	
	*p*			0.0134*		0.0768		0.0021*		0.3299	

Prev: Prevalence; Caries exp: caries experience; IQR: Interquartile range

DMFT: Decayed, Missed, filled teeth

D: decayed; M: missed; F: filled

For comparison between DMFT, D, M, F, and sociodemographic, oral health determinants Median test was used

For comparison between DMFT, D, M, F, and number of household members Kruskal-Wallis test was used

*Relationship significant less than 5%

The most affected age group, according to the DMFT index and each of its components, was 35–45 years. Regarding the region, it was found that women who lived in the Orinoquia and Amazonia regions had the highest frequency of caries when compared to the other regions. Women who were working were also the most affected compared to those who were unemployed. The number of household members was found to be associated with the DMFT index and with the closed component, showing that the greater the number of members (three or more) was, the greater the frequency of this index compared to households with fewer members.

When evaluating the factors potentially associated with the DMFT index in pregnant women, it was found that age was an important factor. Pregnant women aged 35–45 years had 2.41 times more DMFT (CR = 2.41; 95% CI: 2.06–2.80) compared to women younger than 25, and this remained constant when stratified analysis was performed (Table 3).

**Table 3:** Association between sociodemographic, context, individual lifestyles and behaviors, and dental care lifestyles with number of DMFT. Negative binomial regression

Variables		Final Model
		CR (IC 95%)
Age	15–24	1
	25–34	**1.69 (1.53–1.86)**
	35–45	**2.41 (2.06–2.80)**
Region	Atlantica	1
	Oriental	**1.21 (1.03–1.42)**
	Central	0.92(0.77–1.09)
	Pacifica	1.04 (0.88–1.22)
	Bogotá	1.12 (0.96–1.33)
	Orinoquia-Amazonia	**1.19 (1.02–1.39)**
Race / Ethnicity	White	1
	Mongrel	0.95(0.85–1.07)
	Black / Brown	**0.77 (0.64–0.91)**
	Other ethnicities	1.14 (0.95–1.38)
	don’t know / not defined	0.92 (0.79–1.08)
Reasons for last dental visit	Prevention / Control	1
	Never	**0.66 (0.47–0.93)**
	Emergency	1.08 (0.89–1.30)
	Treatment	**1.26 (1.11–1.44)**
	Antenatal care	1.06 (0.95–1.21)
Trimester of referral	First	1
	No referral/no appointment	0.93 (0.84–1.04)
	Second	**0.87 (0.76–0.98)**
	Third	0.86 (0.66–1.10)

Bold: relationship significant at the 5% level

CR: Count rate

AIC: 5838 BIC: 5937

Final Model: adjusted for age group, context, individual lifestyles and behaviors and dental lifestyle

Pregnant women who live in the eastern region and Orinoquia and Amazonia regions had 1.21 to 1.19 times more DMFT than those who live in the Atlantic region (CR = 1.21 95% CI 1.03–1.42 and CR = 1.19 95% CI 1.02–1.39, respectively). In addition, pregnant women who belonged to black/brown had 0.77 times less DMFT than those who are white (CR = 0.77 95% CI 0.64–0.91). (Table 3)

When the last dental consultation was due to treatment women had 1.26 time more DMFT compared with those who attended a check-up (CR = 1.26 95% CI 1.11–1.44). Pregnant women who were referred for a dental consultation in the second trimester had 0.87 times less DMFT than those who were referred in the first trimester (CR = 0.87 95% CI 0.76–0.98). Likewise, women who did not attend a dental check-up had 0.66 times less DMFT compared with those who attended a dental check-up (CR = 0.66 95% CI 0.47–0.93). (Table 3)

When the index is broken down into components, for the Decay component (D), pregnant women ages 35–45 had 1.46 times a greater number of decayed compared with women ages 15–24 (CR = 1.46 95% CI 1.09–1.95). Pregnant women who belonged to the subsidized insurance population, had 1.42 times a greater number of decayed teeth compared with those who belong to the contributory insurance population (CR = 1.42 95% CI 1.17–1.72). The degree of education was also associated with a higher count of teeth with caries; women who have a secondary education level had 0.78 times less number of decay teeth than those who have a primary education or less (CR = 0.78 95% CI 0.61–0.99). Regarding oral hygiene habits, it was found that women who do not use dental floss had 1.76 times a higher count of decayed teeth compared with those who floss more than twice a day (CR = 1.76 95% CI 1.29–2.39). (Table 4)

**Table 4:** Association between sociodemographic, context, individual lifestyles and behaviors and dental care lifestyles with number of Decay. Negative binomial regression

Variables		Final Model
		CR (IC 95%)
Age	15–24	1
	25–34	1.16 (0.97–1.37)
	35–45	**1.46 (1.09–1.95)**
Region	Atlantica	1
	Oriental	**0.65 (0.49–0.87)**
	Central	**0.52 (0.39–0.71)**
	Pacifica	**0.63 (0.48–0.83)**
	Bogotá	0.79 (0.61–1.04)
	Orinoquia-Amazonia	**0.68 (0.52–0.89)**
Race / Ethnicity	White	1
	Mongrel	0.93 (0.76–1.14)
	Black / Brown	0.82 (0.61–1.11)
	Other ethnicities	1.16 (0.84–1.60)
	don’t know / not defined	0.77 (0.59–1.02)
Health Insurance System	Contributory	1
	Subsidiary	**1.42 (1.17–1.72)**
Education Level	Primary	1
	Secondary school	**0.78 (0.61–0.99)**
	Technical	**0.64 (0.48–0.87)**
	University degree or more	**0.43 (0.29–0.63)**
Dental floss frequency	Twice or more a day	1
	No	**1.76 (1.29–2.39)**
	Not every day	1.38 (0.97–1.95)
	Once a day	1.24 (0.88–1.77)

Bold: relationship significant at the 5% level

CR: Count rate

AIC: 3653 BIC: 3752

Final Model: adjusted for age group, context, individual lifestyles and behaviors and dental lifestyle

Regarding filled teeth (F), it was again observed that pregnant women ages 35–45 had 2.46 times a greater number of filled teeth than those ages 15–24 (CR = 2.46 CI95. % 1.93–3.13). Pregnant women who attended their last dental consultation for treatment had 1.39 times greater number of filled teeth those who attended by revision (CR = 1.39 95% CI 1.15–1.67). Pregnant women with primary education or less, had 0.65 times less filled teeth than those with a university degree or more (CR = 0.65 95% CI 0.48–0.87). (Table 5)

**Table 5:** Association between sociodemographic, context, individual lifestyles and behaviors and dental care lifestyles with number of Filled. Negative binomial regression

Variables		Final Model
		CR (IC 95%)
Age	15–24	1
	25–34	**1.87 (1.62–2.15)**
	35–45	**2.48 (1.96–3.12)**
Region	Atlantica	1
	Oriental	**1.90 (1.49–2.42)**
	Central	**1.43 (1.11–1.84)**
	Pacifica	**1.58 (1.25-2.00)**
	Bogotá	**1.59 (1.26–2.01)**
	Orinoquia-Amazonia	**1.82 (1.44–2.30)**
Race / Ethnicity	White	1
	Mongrel	1.04 (0.88–1.22)
	Black / Brown	**0.70 (0.54–0.92)**
	Other ethnicities	1.11 (0.84–1.47)
	don’t know / not defined	1.04 (0.83–1.31)
Education Level	Primary	1
	Secondary school	**1.41 (1.14–1.75)**
	Technical	**1.72 (1.34–2.20)**
	University degree or more	**1.66 (1.26–2.19)**
Reasons for last dental visit	Prevention / Control	1
	Never	0.00 (0.00- $\infty$)
	Emergency	0.94 (0.71–1.25)
	Treatment	**1.36 (1.13–1.63)**
	Antenatal care	1.10 (0.93–1.30)
Dental floss frequency	Twice or more a day	1
	No	**0.76 (0.60–0.95)**
	Not every day	1.06 (0.82–1.36)
	Once a day	1.01 (0.79–1.29)

Bold: relationship significant at the 5% level

CR: Count rate

AIC: 4804 BIC: 4918

Final Model: adjusted for age group, context, individual lifestyles and behaviors and dental lifestyle

For the missing component (M), pregnant women who attended the last dental consultation due to the urgency had 1.65 times more missing teeth than those who attended consultation for revision (CR = 1.65 95% CI 1.08–2.51). Women who attended dental consultation for treatment had 1.60 times more missing teeth than those who attended for revision (CR = 1.60 95% CI 1.20–2.14). Pregnant women who were in medical treatment other than prenatal control had 0.57 times less count of missing teeth than those who were in prenatal control (CR = 0.57 95% CI 0.33–0.96). (Table 6)

**Table 6:** Association between sociodemographic, context, individual lifestyles and behaviors and dental care lifestyles with number of Missing. Negative binomial regression

Variables		Final Model
		CR (IC 95%)
Age	15–24	1
	25–34	**3.28 (2.63–4.13)**
	35–45	**8.88 (6.42–12.2)**
Race / Ethnicity	White	1
	Mongrel	0.81 (0.63–1.05)
	Black / Brown	0.78 (0.53–1.13)
	Other ethnicities	**1.62 (1.10–2.41)**
	don’t know / not defined	1.02 (0.72–1.44)
Reasons for last dental visit	Prevention / Control	1
	Never	**0.15 (0.04–0.55)**
	Emergency	**1.65 (1.08–2.51)**
	Treatment	**1.60 (1.20–2.14)**
	Antenatal care	1.23 (0.93–1.63)
Type of medical treatment	Antenatal care	1
	No treatment	0.81 (0.64–1.03)
	Other	**0.53 (0.30–0.91)**
Dental floss frequency	Twice or more a day	1
	No	**2.06 (1.43–2.97)**
	Not every day	1.31 (0.85-2.00)
	Once a day	**1.60 (1.06–2.44)**

Bold: relationship significant at the 5% level

CR: Count rate

AIC: 2637 BIC: 2721

Final Model: adjusted for age group, context, individual lifestyles and behaviors and dental lifestyle

## Discussion

This study, using data from the last population-based study of pregnant women in Colombia in 2014, has provided knowledge on the association between oral health, specifically dental caries, and the social determinants of oral health in pregnant women in Colombia. This study has as strengths the population design and representativeness of the study sample. The findings of this study show a high prevalence of dental caries in the evaluated pregnant women, observing that 59% have at least one tooth with dental caries, 70.9% have at least one tooth filled and 38.7% have lost at least one tooth due to caries. The age group that presented a higher prevalence (29.3%) and caries experience (43.3%) was 15–24 years, and these prevalences declined with age.

For the DMFT index, the median for this group is 4, increasing to 11 in the 35–45 age group. This is explained by the fact that the D component (decay) is higher in the younger population and lower in the older population. Concerning components F (filled) and M (missing), it is higher in the older population, with component F being the highest for this population, which indicates that this group regularly attends oral health services. The high count of filled teeth found in this study agrees with that found in the study by Thomas et al. 2008 [8] in Australia, Vera-Delgado et al. 2010 [9] in Spain, and Misrachi et al. 2009 [10] in Chile, where this DMFT component was the highest, unlike that found by Deghatipour et al. 2019 [11] in Iran, Gutpa et al. 2016 [12] and Ingle et al. in 2014 [13] in India, and Karunachandra et al. 2012 [14] in Sri Lanka. The latter are middle-income countries unlike medium-high-income countries Colombia and Iran, which may be the cause of the differences in access to health services and oral care patterns between countries with different levels of income. In addition, significant differences were found in the DMFT index according to the region of origin of the pregnant women, where it is observed that the region is the explanation for the differences found in the index, possibly due to socioeconomic and cultural differences between regions. This situation is similar to that found by Deghatipour et al. 2019 [11], where there were differences in the DMFT index according to the region of origin.

Regarding education, it was found that there was a relation between the level of education of the mother and the caries experience, observing that at a lower level of education, there was an increase in the count of decayed teeth, unlike what happens with the component of filled teeth, where having a lower level of education decreases the number of filled teeth. This is in agreement with the study by Deghatipour et al. in 2019 [11], who found a lower rate of caries and a greater number of fillings in pregnant women with an education level greater than 12 years of study. These results are similar to those found in this study where pregnant women whose maximum education level was a university degree have fewer decayed teeth and a greater number of filled teeth (p < 0.000). Kateeb et al. in 2018 [15] found that education is an important and significant factor in the experience of caries, observing that pregnant women who had a university degree had lower DMFT values than those who had completed high school, unlike this study where no difference was found in the DMFT (p = 0.392) according to education. This may be because in our population the number of teeth affected, treated, and lost due to caries is greater, which affects the index and therefore no difference can be seen in the index according to educational level. It should be noted that when it is evaluated in each of its components, educational level is an important and significant factor. Vera-Delgado et al. 2010 [9] observed that the more years of study the women had, the lower the number of active dental caries (p = 0.000), and the greater the number of teeth filled (p = 0.001).

In this study, 78.5% of pregnant women had attended a dental consultation within the previous six months, and the reason for the dental consultation was prenatal care in 40.3%. Regarding the association between the reason for consultation and the DMFT, it was found that attending for treatment increases the index count in this population. The same occurs with the count of filled and lost teeth where in the latter it is added to attend for emergencies, which could suggest that pregnant women come to the consultation with advanced caries lesions that lead to fillings and in some cases to tooth loss. Krüger et al. 2015 [16] found that of the 105 pregnant women evaluated in their study, 33.4% sought dental care, and the reason for such care in 47.6% was pain. These findings are similar to those of Ibrahim et al. 2012 [17] who found that 94.1% of pregnant women went to dental consultation for treatment and 5% for preventive or control reasons. Deghatipour et al. 2019 [11] observed that half of the pregnant women evaluated had not attended a dental consultation in the previous year, and in these women, a lower count of missing teeth was observed. These results are similar to those found in this study, where not having gone to the dentist decreased the count of teeth lost due to caries. This result may be because 35% of the pregnant women were not referred for dental check-ups during prenatal check-ups, and those who were referred (7.4%) did not obtain an appointment, which led to unresolved dental problems. This situation is similar to that found in the study by Boggess et al. 2010 [18] and Singhal et al. 2014 [19], who found that 25% and 48.2% of the pregnant women evaluated reported having attended the dentist for control during pregnancy, respectively, and 74% and 51.7%, respectively, reported not having attended for maintenance during pregnancy. The main reason subjects in the Boggess study gave was economic, and 7% reported not going because they were told not to go during pregnancy.

In the present study, it was found that 64.3% of pregnant women brushed their teeth three times a day or more, and 56.3% did not use dental floss; the latter was found to be associated with an increase in the DMFT index count of decayed and missing teeth as opposed to filled teeth. These findings indicate the importance of the use of dental floss as part of oral hygiene. In addition, these findings call attention to the fact that greater education should be carried out for pregnant women concerning oral hygiene and its importance because if they do not take care of themselves, they will probably not take care of their children’s oral hygiene. Okada et al. 2002 [20] found that the oral behaviors of the parents affected the oral behaviors of the children (p < 0.001) and the component of decayed teeth of children (p < 0.005) shows the importance of reinforcing the education of the parents to have a positive effect on subsequent generations.

Among the limitations of this study is that it is a cross-sectional study that does not allow establishing causal relationships since both the exposure and the outcome occurred before the start of the study, the directionality cannot be established, which means that whether the risk factor occurred before the outcome cannot be established.

Another limitation of the study is that the medical history was self-reported, which can lead to biases such as over/underreporting or recall bias. This study, being a national study, has particularities such as the Health Regime that are not comparable with other countries. Additionally, since the study was conducted in 2014, the age of the data may not represent the current reality.

## Conclusions

This study using data from the last population-based study conducted in Colombia in 2014 found a caries prevalence of 59% in pregnant women, which was higher among younger women. Concerning the DMFT index, it was observed that it is higher in the group of pregnant women ages 35–45. The maximum education level is an important factor associated with dental caries and filled teeth, so the role of oral health education and encouraging pregnant women to attend dental check-ups during this stage is key to reducing the prevalence of caries not only in this population but also in their children. It is also important to train doctors and nurses who perform prenatal check-ups to refer and encourage pregnant women to ask for and attend dental check-ups. It is of vital importance to develop educational strategies in oral hygiene so that these pregnant women know and reinforce their knowledge, considering that only 64.3% of this population brushes their teeth three times or more a day and 56.3% do not use dental floss. Their oral hygiene routine and behaviors will impact not only their health but also that of their children.

### Electronic supplementary material

Below is the link to the electronic supplementary material.

Supplementary Material 1

## Data Availability

The data that support the findings of this study are available from Ministry of Health, but restrictions apply to the availability of these data, which were used under license for the current study, and so are not publicly available. Data are however available from the corresponding author upon reasonable request and with permission of Ministry of Health.
