# Supplementary material for: Prevalence of dental caries in pregnant Colombian women and its associated factors
Source: BMC Oral Health. 2023 Oct 24;23:793. doi: 10.1186/s12903-023-03419-8 (PMC10598961; doi:10.1186/s12903-023-03419-8)
Supplement: Supplementary file 1 — Supplementary Material 1 [file 12903_2023_3419_MOESM1_ESM.docx]

Supplementary Table 1. Variable description

| Question | | Variable | Measurement categories | |
| --- | --- | --- | --- | --- |
| *Sociodemographic characteristics* | | | | |
| What is the date of birth and age in completed years? | Age group | | | 15-24 |
|  |  |  |  | 25-34 |
|  |  |  |  | 35-45 |
| How many months pregnant are you? | Trimester of pregnancy | | | First |
|  |  |  |  | Second |
|  |  |  |  | Third |
| *Context* | | | | |
| Region where the pregnant woman lives? | Region | | | Atlántica |
|  |  |  |  | Oriental |
|  |  |  |  | Central |
|  |  |  |  | Pacifica |
|  |  |  |  | Bogotá |
|  |  |  |  | Orinoquia - Amazonia |
| What is your current marital status? | Marital status | | | Married or living with partner |
|  |  |  |  | Not married or not living with partner |
| With which of the following ethnic groups do you identify? | Race / Ethnicity | | | White |
|  |  |  |  | Mongrel |
|  |  |  |  | Black / Brown |
|  |  |  |  | Other ethnicities |
|  |  |  |  | don't know / not defined |
| What health plan are you affiliated with? | Health Insurance System | | | Contributory |
|  |  |  |  | Subsidiary |
| In which of these ranges does the household's monthly income fall? | Household Income | | | < 1 monthly salary |
|  |  |  |  | 1-2 monthly salaries |
|  |  |  |  | > 2 monthly salaries |
| What is the highest educational level achieved by you? | Education Level | | | Primary |
|  |  |  |  | Secondary school |
|  |  |  |  | Technical |
|  |  |  |  | University degree or more |
| Do you currently work? | Job | | | Yes |
|  |  |  |  | No |
| In your home, is the water supply through? | Water supply | | | Aqueduct with constant supply |
|  |  |  |  | Aqueduct with intermittent supply |
|  |  |  |  | Other water sources |
| What is the area where the pregnant woman lives? | Living area | | | Rural |
|  |  |  |  | Urban |
| What type of housing do you live in? | Type of housing | | | House |
|  |  |  |  | Apartment |
|  |  |  |  | Other type of housing |
| How many people are members of this household? | Number of household members | | | Mean (SD) |
| When was the last time you went to the dentist? | Last dental visit | | | Never |
|  |  |  |  | More than 2 years |
|  |  |  |  | Between 6 months and < 1 year |
|  |  |  |  | Less than 6 months ago |
| *Individual lifestyles and behaviors* | | | | |
| What was the reason you consulted the dentist at the last visit? | Reasons for last dental visit | | | Never |
|  |  |  |  | Emergency |
|  |  |  |  | Treatment |
|  |  |  |  | Prevention / Control |
|  |  |  |  | Antenatal care |
| Where did you receive your last dental visit? | Dental care place | | | Never |
|  |  |  |  | Health center |
|  |  |  |  | EPS |
|  |  |  |  | Private practice |
| Has the health service you use referred you to the  dentist? When they referred you, did they give you the appointment? | Referral to the dentist | | | No |
|  |  |  |  | Referred / no appointment |
|  |  |  |  | Referred / appointment |
| In what month of the pregnancy did you go to the appointment with the dentist? | Trimester of referral | | | No referral/no appointment |
|  |  |  |  | First |
|  |  |  |  | Second |
|  |  |  |  | Third |
| Are you currently undergoing medical treatment? | Currently undergoing medical treatment | | | Yes |
|  |  |  |  | No |
| What medical treatment(s) are you currently undergoing? | Type of medical treatment | | | No |
|  |  |  |  | Antenatal care |
|  |  |  |  | Other |
| *Dental care lifestyles* | | | | |
| How many times do you brush your teeth? | Toothbrushing frequency | | | No |
|  |  |  |  | 1 day |
|  |  |  |  | 2 day |
|  |  |  |  | $\geq$ 3 day |
| How much toothpaste to use for brushing? | Amount of toothpaste | | | No |
|  |  |  |  | ¼ brush |
|  |  |  |  | ½ brush |
|  |  |  |  | ¾ brush |
| How often do you floss? | Dental floss frequency | | | All brush |
|  |  |  |  | No |
|  |  |  |  | Not every day |
|  |  |  |  | Once a day |
